# Reply to Kataoka, N.; Imamura, T. How to Improve Clinical Outcomes in Patients with Tachycardia-Induced Cardiomyopathy. Comment on “Katz et al. Long-Term Outcomes of Tachycardia-Induced Cardiomyopathy Compared with Idiopathic Dilated Cardiomyopathy. *J. Clin. Med.* 2023, *12*, 1412”

**DOI:** 10.3390/jcm12185849

**Published:** 2023-09-08

**Authors:** Moshe Katz, Amit Meitus, Michael Arad, Anthony Aizer, Eyal Nof, Roy Beinart

**Affiliations:** 1 Sheba Medical Center, Ramat Gan 5266202, Israel; michael.arad@sheba.health.gov.il (M.A.); eyalnof.dr@gmail.com (E.N.); beinart.roy@gmail.com (R.B.); 2 School of Medicine, Tel-Aviv University, Tel Aviv 6997801, Israel; amymeitus@gmail.com; 3 NYU Grossman School of Medicine, New York, NY 10016, USA; anthony.aizer@nyulangone.org

In a letter to the editor titled “How to improve clinical outcomes in patients with tachycardia-induced cardiomyopathy”, Dr. Naoya Kataoka and Dr. Teruhiko Imamura [1] have raised several concerns regarding the paper titled “Long-term outcomes of tachycardia induced cardiomyopathy compared with idiopathic dilated cardiomyopathy” [2]. 

The first concern was about the definitions of tachycardia-induced cardiomyopathy (TICMP) and idiopathic dilated cardiomyopathy (IDCM). In the paper, we used acceptable and common definitions as described in the literature. Patients were classified as having TICMP if they presented with heart failure secondary to arrhythmia without any other apparent causes for cardiomyopathy and showed an improvement of at least 15% in LVEF after rhythm control or rate control within 6 months [3,4,5]. Others define TICMP as the presence of reversible left ventricular dysfunction solely due to increased ventricular rates [6] and use a broader term, arrhythmia-induced cardiomyopathy (AIC), for patients with persistent fast ventricular rate or for patients with atrial or ventricular ectopy coexisting with cardiomyopathy. They claim that arrhythmia (e.g., atrial fibrillation) may lead to non-ischemic dilated cardiomyopathy by promoting dyssynchrony in the absence of tachycardia, and through the elimination of arrythmia, it is expected that left ventricular (LV) function will improve. To the point, in our paper we used the term TICMP to describe patients with any arrhythmia that resulted in cardiomyopathy, and not only patients with LV dysfunction due to increased ventricular rates. Nevertheless, only one patient out of four had a high burden of ventricular ectopy without clear sustained tachycardia (Table S1 [2]). During follow-up, four patients in the TICMP received cardiac resynchronization therapy (CRT) (Table S2 [2]). Three out of the four patients underwent CRT implantation for rate control due to unsuccessful rhythm or medical rate control. The fourth patient underwent CRTD implantation 6 years after the index hospitalization. This patient’s LVEF improved significantly after the index hospitalization, and during follow-up developed cardiomyopathy with secondary malignant arrhythmia. Hence, all these patients were appropriately classified in the TICMP group. In the letter to the editor, the authors suggested to define TICMP as those with improvements in LV systolic function following the elimination of arrhythmias without using any devices that affect ventricular function. The authors assume that the elimination of arrhythmias is always feasible. However, some patients have arrhythmias that are hard to control and different strategies have failed to control their arrhythmia. These patients can earn from the pace and ablate strategy as was performed for three patients in our cohort. Regarding the IDCM group, IDCM was defined as LVEF ≤ 50% at presentation in the absence of any of the following conditions: ischemia, uncontrolled hypertension (>160/100), severe valvular disease, congenital heart disease, toxic exposure (chemotherapy/alcohol consumption etc.), metabolic etiologies (nutritional deficiencies, endocrinopathy etc.), or tachyarrhythmia [7]. In the IDCM cohort, five patients had atrial fibrillation and atrial flutter at presentation (Table S1 [2]). The association between atrial fibrillation/flutter and cardiomyopathy occurs in up to 30% of patients with inherited cardiomyopathies, and atrial fibrillation is often the first presentation of idiopathic cardiomyopathy. Atrial fibrillation may lead to superimposed cardiomyopathy in patients with idiopathic cardiomyopathy (arrhythmia-mediated) [5], which does not preclude an underlying cardiomyopathy. A lack of improvement or mild improvement in LVEF (less than 15%) after rhythm or rate control supports the diagnosis of underlying cardiomyopathy. It is unlikely that rhythm or rate control will not lead to significant improvement in LVEF when arrhythmia is the patient’s sole problem. 

A second concern was about the high mortality of patients in the TICMP group. During a median follow-up time of 6.43 years [IQR 5.2–8.2], 14 TICMP patients (22%) died (Table 3 [2]). In the CASTLE-AF trial, during a median follow-up of approximately 3 years, 13.4% of the patients died from any cause in the ablation group and 25% died in the medical-therapy group. In the CABANA trial, during a median follow-up time of approximately 4 years, all-cause mortality in patients with atrial fibrillation and heart failure was lower than in our cohort as well (6.1% in the ablation arm versus 9.3% in the drug therapy arm) [8]. The difference in mortality between our cohort and the above randomized controlled trials (RCTs) may stem from the differences in follow-up time, selection bias, treatment strategy, and follow-up. A higher mortality is expected in longer follow-ups, as seen in our cohort. In addition, our cohort is real-world data of all comers with regular follow-up. Randomized control trials have stricter follow-ups and can therefore address any change in a patient’s medical condition more rapidly and address the recurrence of arrhythmia earlier. Moreover, patients enrolled in RCTs are usually healthier than real-world patients. Therefore, a better prognosis is expected in RCTs. Finally, early intervention with catheter ablation may lead to improved survival in patients with TICMP. In our cohort, only a few patients received an early invasive intervention for their arrhythmia. As mentioned in the paper, implementation of the guidelines’ recommendations of an early invasive strategy, together with tight patient monitoring, could lead to a reduction in clinical events and potentially improve prognosis in selected patients. 

The third concern was about guideline-directed medical therapy. A major change was made in recommendations regarding GDMT in the last heart failure ESC and ACC guidelines [9,10]. From 2016 until recently, the main drugs to treat patients with symptomatic heart failure with reduced ejection fraction were beta blockers, angiotensin-converting enzyme inhibitor (ACEI)/angiotensin receptor blocker (ARB), mineralocorticoid receptor (MR), and angiotensin receptor neprilysin inhibitor (ARNI) [11]. In the new guidelines, the sodium–glucose cotransporter-2 inhibitors (SGLT2i) were added. In our cohort, most of the patients were treated according to previous guidelines. At discharge, 85% of IDCM patients received beta blockers versus 72% of TICMP patients; 91% of IDCM patients received ACEI/ARB versus 75% of TICMP patients; and 45% of TICMP patients received MR versus 33% of TICMP patients. As acknowledged in the study limitations, our cohort included patients who were enrolled between March 2007 and June 2017. At that time, more conservative treatment strategies were adopted, and the newer heart failure drugs (SGLTi and ARNI) were not commonly available. Therefore, we did not collect data on SGLTi and ARNI, which were only available after 2016. Adding ARNI and SGLT2i to the medical regimen may improve survival and reduce hospitalization for worsening heart failure in both groups (TICMP and IDCM).
